# Identification and expression analyses of the alanine aminotransferase (AlaAT) gene family in poplar seedlings

**DOI:** 10.1038/srep45933

**Published:** 2017-04-05

**Authors:** Zhiru Xu, Jing Ma, Chunpu Qu, Yanbo Hu, Bingqing Hao, Yan Sun, Zhongye Liu, Han Yang, Chengjun Yang, Hongwei Wang, Ying Li, Guanjun Liu

**Affiliations:** 1 State Key Laboratory of Tree Genetics and Breeding (Northeast Forestry University), School of Forestry, Northeast Forestry University, Harbin 150040, China; 2 College of Life Science, Northeast Forestry University, Harbin 150040, China; 3 School of Forestry, Northeast Forestry University, Harbin 150040, China; 4 Key Laboratory of Saline-alkali Vegetation Ecology Restoration in Oil Field (SAVER), Ministry of education, Alkali Soil Natural Environmental Science Center (ASNESC), Northeast Forestry University, Harbin 150040, China

## Abstract

Alanine aminotransferase (AlaAT, E.C.2.6.1.2) catalyzes the reversible conversion of pyruvate and glutamate to alanine and α-oxoglutarate. The *AlaAT* gene family has been well studied in some herbaceous plants, but has not been well characterized in woody plants. In this study, we identified four alanine aminotransferase homologues in *Populus trichocarpa,* which could be classified into two subgroups, A and B. *AlaAT3* and *AlaAT4* in subgroup A encode AlaAT, while *AlaAT1* and *AlaAT2* in subgroup B encode glutamate:glyoxylate aminotransferase (GGAT), which catalyzes the reaction of glutamate and glyoxylate to α-oxoglutarate and glycine. Four *AlaAT* genes were cloned from *P. simonii* × *P. nigra. PnAlaAT1* and *PnAlaAT2* were expressed predominantly in leaves and induced by exogenous nitrogen and exhibited a diurnal fluctuation in leaves, but was inhibited in roots. *PnAlaAT3* and *PnAlaAT4* were mainly expressed in roots, stems and leaves, and was induced by exogenous nitrogen. The expression of *PnAlaAT3* gene could be regulated by glutamine or its related metabolites in roots. Our results suggest that *PnAlaAT3* gene may play an important role in nitrogen metabolism and is regulated by glutamine or its related metabolites in the roots of *P. simonii* × *P. nigra*.

Nitrogen is an essential nutrient element for plant growth. The use of nitrogen directly affects plant growth and development, biomass and grain yield. Poplar has great potential applications in CO_2_ mitigation and biofuel production^1^, and is perhaps more often used for pulpwood and nowdays as a biomass crop^2^. Poplar can exchange N with the environment by opening or closing the N cycle^3^, and thus plays a critical role in the ecosystem N cycle^3^,^4^. However, usually acting as a shelter forest, poplar is often established on marginal lands where the soil N is limited^5^. To achieve sustainable high productivity and decrease N fertilization, it is important to obtain a better understanding of the molecular regulatory mechanisms of N utilization.

Nitrate (NO_3_^−^) and ammonium (NH_4_^+^) are the main sources of inorganic N in the soil. They can be absorbed by roots through at least two transport systems^6^,^7^. NO_3_^−^ is transported into roots by nitrate transporters (NRT), and then reduced to NH_4_^+^ by nitrate reductase (NR) and nitrite reductase (NiR). NH_4_^+^ is transported into roots by ammonium transporters (AMT), assimilated into glutamine and glutamate through the glutamine synthetase (GS) and glutamate synthase (GOGAT) cycle, and further incorporated into other amino acids by aminotransferase^8^. NH_4_^+^ and NO_3_^−^ have different effects on plant growth, as the pH of the medium is reduced after NH_4_^+^ is absorbed and increased after NO_3_^−^ is absorbed, which affects the availability of other nutrients^9^. When NH_4_^+^ is supplied as the sole N source, many plants showed negative effects, such as reduced leaf area, relative growth rate and dry matter yield^10^,^11^,^12^. In contrast to NH_4_^+^, the presence of NO_3_^−^ stimulated the germination of dormant seeds of *Arabidopsis thaliana*^13^, regulated shoot-root allocation in tobacco and floral induction in *A. thaliana*^14^,^15^ and inhibited root growth of maize^16^. Many *Populus* species showed better growth on NO_3_^−^ than on NH_4_^+^^3^, but some authors have reported a preference for NH_4_^+^^17^. Because different N forms have different effects on plant growth and metabolism, the expression of related plant genes might be altered. It is therefore necessary to study the effects of N forms on the expression of genes in N metabolism.

Alanine aminotransferase (AlaAT, E.C.2.6.1.2) is a pyridoxal phosphate- dependent enzyme that catalyses the reversible conversion of alanine and α-oxoglutarate to pyruvate and glutamate. AlaAT is widely distributed in various plant tissues and organs. It is found to be active not only in leaves, roots and flowers^18^, but also in other tissues, such as those of fruit^19^, as well as the inner endosperm tissues of developing rice seeds^20^. The diverse distribution of AlaAT suggests that it may play important roles in the life cycle of plants. Previous research on AlaAT has mainly focused on its role in hypoxic conditions, which has been characterized in *Hordeum vulgare, Medicago truncatula* and *Arabidopsis thaliana*^21^,^22^,^23^. *AlaAT* transcript levels could be induced by hypoxia^22^,^24^,^25^,^26^,^27^. During hypoxia condition induced by waterlogging, AlaAT linked glycolysis and the tricarboxylic acid cycle in *Lotus japonicus*^28^. AlaAT is a limiting factor in alanine synthesis under low-oxygen conditions; the primary role of AlaAT1 is to break down alanine when it is in excess in *A. thaliana*^23^.

Nevertheless, we are more interested in the role of AlaAT in nitrogen and carbon metabolisms. *AlaAT* expression is not only regulated by hypoxia but also by light and N^49^,^30^. AlaAT has been widely studied using genetic engineering methods in recent years. Over-expression of a barley *AlaAT* in *Brassica napus* (canola) and *Oryza sativa* (rice) increased the yield and biomass of the transgenic plants^31^,^32^. The transcriptome of transgenic rice roots and shoots over-expressing alanine aminotransferase under the control of a tissue-specific promoter was not significantly different from that of controls^33^. Sugarcane lines transformed with barley alanine aminotransferase gene driven by rice aldehyde dehydrogenase gene (*OsAnt1*) promoter showed improved N use efficiency compared with untransformed ones in a pot trial under low nitrogen conditions^34^. Recent studies have indicated that the over-expression of *AlaAT* variants other than barley *AlaAT* in *A. thaliana* could further increase the N use efficiency phenotype(s)^35^.

Studies on the *AlaAT* gene family have mainly focused on herbaceous plant species. In *A. thaliana*, four *AlaAT* genes have been cloned, including *AtAlaAT1* and *AtAlaAT2* encoding alanine aminotransferase (E.C.2.6.1.2), and *AtGGAT1* and *AtGGAT2* encoding GGAT (E.C.2.6.1.4) with glutamate:glyoxylate aminotransferase activity^18^,^36^. In *Glycine max*, two subclasses were identified, with each subclass represented by two highly similar members with the same gene structure^37^. Similarly, four *AlaAT*s were found to be expressed in *M. truncatula*^22^. Unlike herbaceous plants, tree species have a long lifespan, long generation times and a perennial woody growth habit^38^. The N nutrition of trees is sustained by seasonal and internal cycling^3^. Studying the molecular regulatory mechanisms of N in poplar has great environmental significance. In the present study, we characterized the *AlaAT* gene family members in *P. trichocarpa*, and cloned them from *P. simonii* × *P. nigra*. We then investigated the expression profile of the *PnAlaAT*s genes by real-time quantitative PCR in leaves, stems and roots of *P. simonii × P. nigra* supplied with different N sources and light levels. Additionally, the regulation of *PnAlaAT3* in roots was studied under treatment with methionine sulfoximine (MSX), an inhibitor of glutamine synthetase.

## Results

### Identification of *AlaAT* genes in *P. trichocarpa*

According to the methods of Wang *et al*.^39^ and Chai *et al*.^40^, the Hidden Markov Model (HMM) profile “PF00155” was searched against the *P. trichocarpa* genome to identify *AlaAT* genes. Four sequences (XM_002315639, XM_002312643, XM_006369021, XM_002304219) located on different chromosomes were found in the *P. trichocarpa* genome. The total length of each of the four sequences was 1446 bp, encoding 481 amino acids (Table 1).

**Figure 1:**
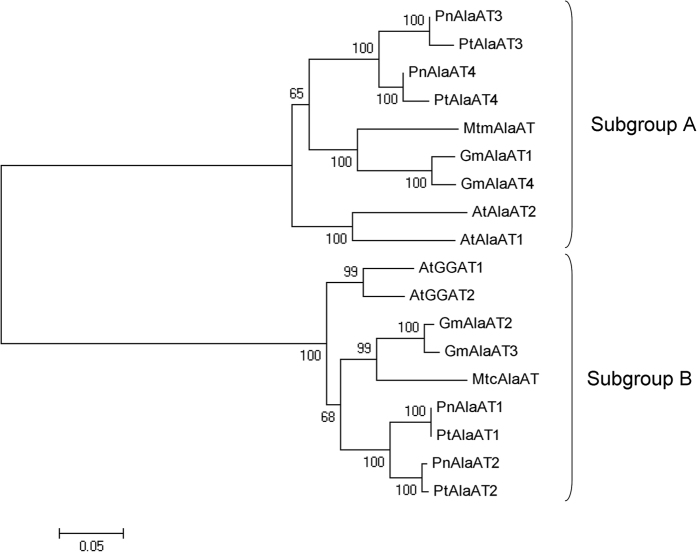
Phylogenetic tree of proteins encoded by AlaAT genes from *A. thaliana, G. max, M. truncatula, P. trichocarpa* and *P. simonii × P. nigra.* Protein sequences were aligned by ClustalW and phylogenetic tree was constructed by MEGA5 using N-J method, with 1000 bootstrap replicates.

**Figure 2:**
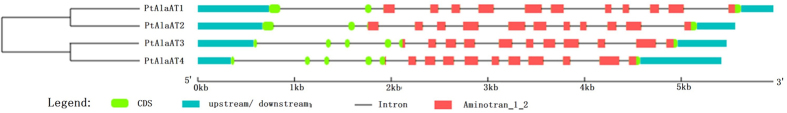
Comparison of the intron and exon structure of the four *AlaAT* genomic sequences identified in *P. trichocarpa*. Green boxes- coding exons, red boxes- coding Aminotran_1_2 exons, black lines- introns. All of the poplar *AlaAT* gene family members had the accordingly conserved Aminotran_1_2 exons.

The *Populus AlaAT* genes had high sequence similarity to the previously characterized *AlaAT* genes from *A. thaliana*^18^, *M. truncatula*^22^ and *G. max*^37^. We aligned the full-length amino acid sequences of the AlaATs with ClustalW (http://www.ebi.ac.uk/Tools/msa/clustalw2/) (Figure S1). A phylogenetic tree was constructed using the neighbor-joining method and Poisson correction model with the MEGA5 software^41^. The phylogenetic tree showed that the AlaAT family was clearly separated into two subgroups, with two *Populus* AlaAT homologues per subgroup (Fig. 1). PtAlaAT1 and PtAlaAT2 were clustered together in subgroup B close to *A. thaliana* AtGGAT1 and AtGGAT2, whereas PtAlaAT3 and PtAlaAT4 were grouped together in subgroup A close to AtAlaAT1 and AtAlaAT2. PtAlaAT1 and PtAlaAT2 shared the same gene structure, as did PtAlaAT3 and PtAlaAT4 (Fig. 2).

### Regulatory regions in the poplar *AlaAT* genes

**Figure 3:**
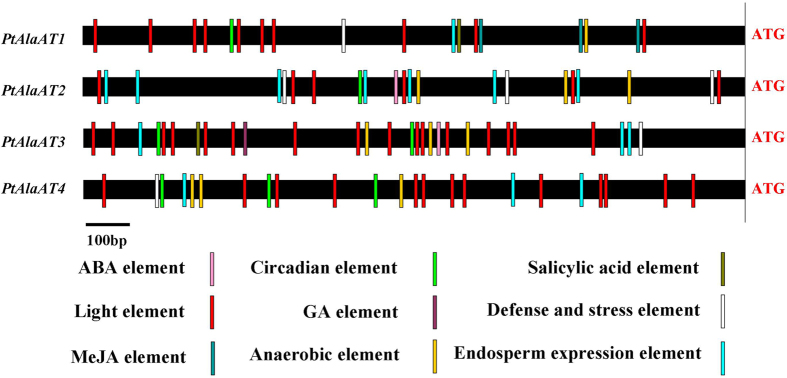
Regulatory regions of the *P. trichocarpa* AlaAT genes. The 5′ upstream regions of AlaAT genes are represented. Regulatory elements conserved in each promter are marked by colours. The position of the ATG is marked on the right.

To get insight into the functions of the *AlaAT* genes in poplar, the putative regulatory elements in their 5′-upstream regions were investigated (Fig. 3). In all four genes, regulatory elements were found to be concentrated in the promoter region about 700–900 bp upstream of the translation initiation site. Abscisic acid (ABA) responsive elements were identified in the promoters of *PtAlaAT2* and *PtAlaAT3*, MeJA-responsive elements were found exclusively in the *PtAlaAT1* promoter, a gibberellin-responsive element (GA element) were found only in the *PtAlaAT3* promoter, and salicylic acid responsive elements were present in the promoters of *PtAlaAT1* and *PtAlaAT3*. All of the promoters contained anaerobic, circadian control, endosperm expression, defense and stress responsive elements, as well as many light-responsive elements, especially the *PtAlaAT3* promoter.

### Cloning the cDNAs of *AlaAT* family genes from *P. simonii* × *P. nigra*

Total RNA from mixed samples of roots, stems and leaves from *P. simonii* × *P. nigra* plants (about 30 cm tall) were reverse transcribed and full-length *AlaAT* cDNAs were amplified using the RT-PCR technique. Four clones (*PnAlaAT1, PnAlaAT2, PnAlaAT3* and *PnAlaAT4*) were obtained. The open reading frames (ORFs) of *PnAlaAT1, PnAlaAT2, PnAlaAT3* and *PnAlaAT4* each encoded proteins of 481 amino acid residues. The cDNA sequences were aligned and the *AlaAT* gene sequences were found to be highly homologous (99%) between *P. simonii* × *P. nigra* and *P. trichocarpa*. The percentage identity (94%) between *PnAlaAT1* and *PnAlaAT2* was highest, followed by that (93%) between *PnAlaAT3* and *PnAlaAT4* (Figure S2). However, the percentage identity between *PnAlaAT1*/*PnAlaAT2* and *PnAlaAT3*/*PnAlaAT4* was low, only about 50%. According to Igarashi *et al*.^18^, the carboxy-terminal tripeptides of PnAlaAT1 (SRL) and PnAlaAT2 (SRL) are conserved peroxisome -targeting signal-like (PTS1-like) sequences.

### Expression analysis of *PnAlaATs* in different organs of *P. simonii* × *P. nigra*

**Figure 4:**
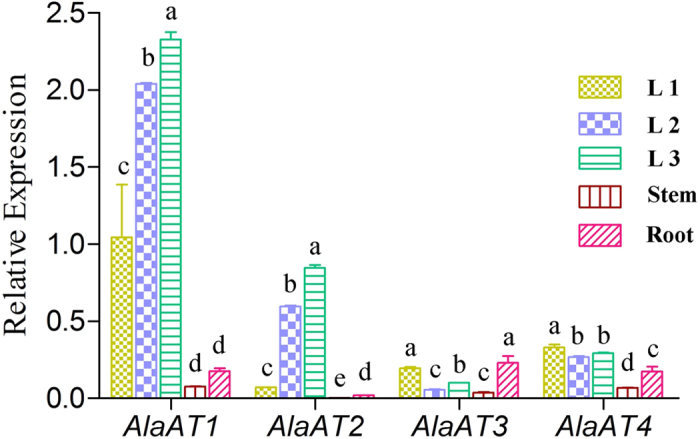
Relative transcript levels of *PnAlaAT* genes in different organs of *P. simonii × P. nigra*. Quantitative RT-PCR was performed using total RNA extracted from leaves, stems and roots of 30 cm tall plants. Results are the mean ± SE of three replicates. (*P* < 0.05). L1: 1st–3rd leaves from the top of the plant; L2: 4th–6th leaves from the top of the plant; L3: 1st–3rd leaves from the bottom of the plant.

Determining the expression of gene family members in different organs provides important information on gene functions. To help characterizing of the functions of the *PnAlaAT* isogenes, their expression profiles were identified by quantitative real-time PCR in roots, stems and leaves. The leaves were divided into three groups to better characterize the expression patterns during leaf development. Four pairs of PCR primers were designed in 3′-or 5′-untranslated regions for specific amplification of each *PnAlaAT* isogene. The result showed (Fig. 4) that *PnAlaAT1* and *PnAlaAT2* were expressed mainly in the leaves, and the expression levels of these two genes in L3 were highest among all the organs. *PnAlaAT3* and *PnAlaAT4* were expressed in all the tested organs. The expression level of *PnAlaAT4* in leaves was higher than in roots, but significantly lower than the levels of *PnAlaAT1* and *PnAlaAT2* in leaves. In roots, the expression level of *PnAlaAT3* was higher than those of *PnAlaAT1* and *PnAlaAT2* in roots.

### Effects of nitrogen sources on *PnAlaAT*s expression in different organs of *P. simonii* × *P. nigra*

**Figure 5:**
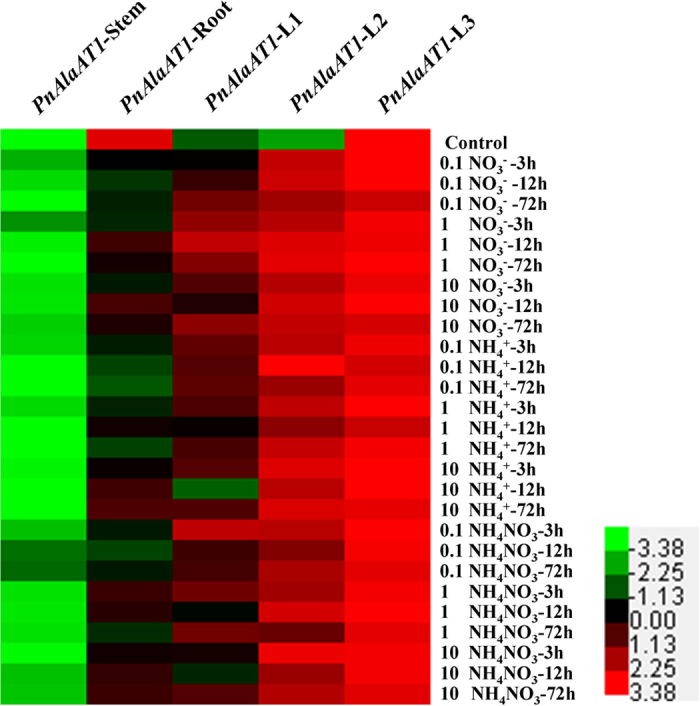
Expression patterns of *PnAlaAT1* gene in different organs under different nitrogen source conditions. Quantitative RT-PCR was performed using total RNA extracted from leaves, stems and roots.

*AlaAT* genes play an important role in the N metabolism process. To investigate the impact of N sources on the *PnAlaAT* genes expresssion, the expression profiles of *PnAlaAT*s were studied by real-time quantitative PCR in leaves, stems and roots grown on different N sources at different concentrations. The result showed that the expression of *PnAlaAT1* in L1 and L2 was induced by different N sources; however, expression in L3 was not induced significantly by exogenous N (Fig. 5). *PnAlaAT1* expression in L1 increased with treatment time under low NO_3_^−^, and reached a peak after 12 h of 1 mM NO_3_^−^ supply. *PnAlaAT1* transcript levels in L1 were lower under 10 mM NO_3_^−^ treatment than that of 1 mM NO_3_^−^. However, *PnAlaAT1* transcript levels did not change significantly in L1 when treated with different concentrations of NH_4_^+^ except 1 mM NH_4_^+^ for 12 h and 10 mM NH_4_^+^ for 72 h. Compared with L1, *PnAlaAT1* abundance in L2 was induced to a high level, irrespective of N form or concentration. Expression of *PnAlaAT1* was the highest in L3 compared with L1 and L2, but was not induced by the N sources. However, *PnAlaAT1* expression in roots was strongly inhibited by different N sources. In stems, *PnAlaAT1* expression levels were low and effectively negligible compared with that in other organs. The expression patterns of *PnAlaAT2* were similar to those of *PnAlaAT1*, but the expression levels in the former were clearly lower than that in the latter (Figure S3).

**Figure 6:**
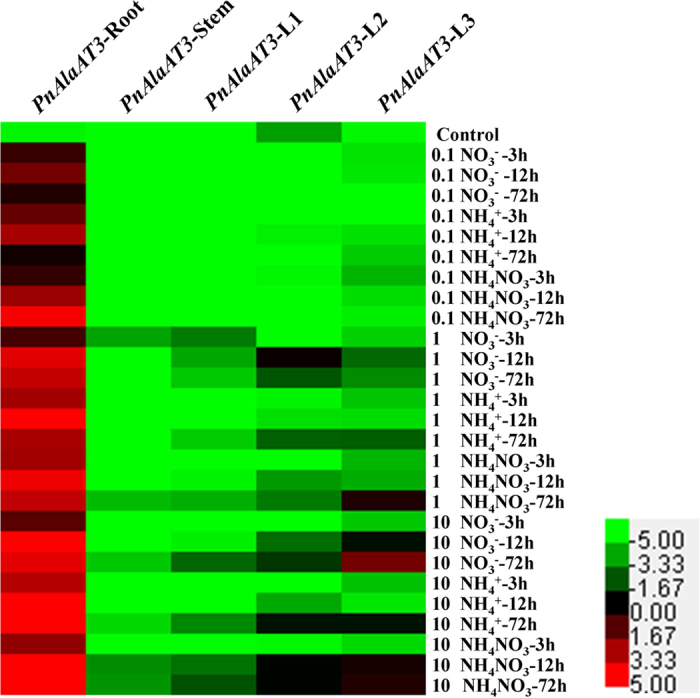
Expression patterns of *PnAlaAT3* gene in different organs under different nitrogen source conditions. Quantitative RT-PCR was performed using total RNA extracted from leaves, stems and roots.

Expression of *PnAlaAT3* gene was strongly induced by exogenous N sources in roots irrespective of N forms (Fig. 6). Notably, the expression levels of *PnAlaAT3* increased by more than 100 times when the plants were treated with 10 mM NH_4_^+^ for 12 h and 72 h in roots. The expression levels of *PnAlaAT3* in leaves were very weak, but increased significantly under high-N treatment. *PnAlaAT3* expression in stems was also induced by exogenous N, but at a level significantly lower than that in roots. In contrast, *PnAlaAT4* was expressed at a negligible level in all tested conditions (Figure S4).

### Effects of the diurnal cycle on *PnAlaAT*s expression in different organs of *P. simonii* × *P. nigra*

**Figure 7:**
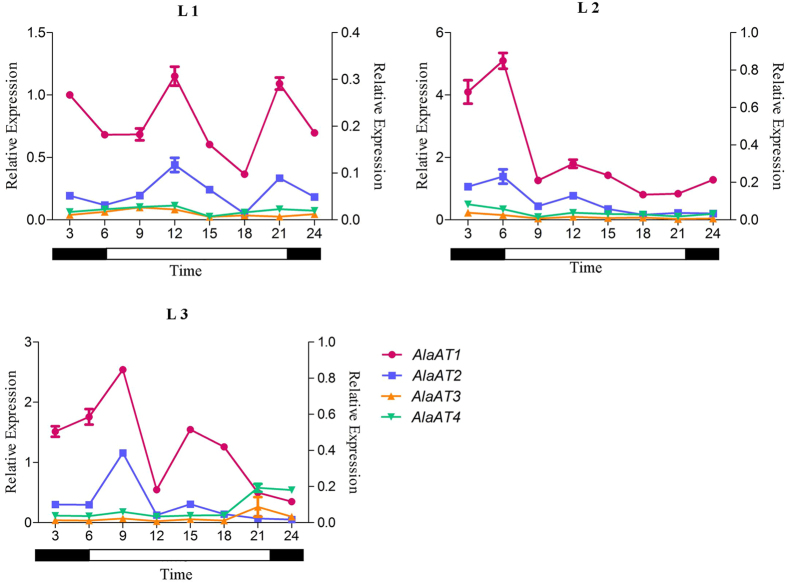
Diurnal fluctuation of expression patterns of *PnAlaAT* genes in leaves. Quantitative RT-PCR was performed using total RNA extracted from leaves. *PnAlaAT1* and *PnAlaAT2* refer to the left Y axis and *PnAlaAT3* and *PnAlaAT4* refer to the right Y axis. Results are the mean ± SE of three replicates.

It is well documented that the transcriptional levels of several plant genes are subject to diurnal control^42^,^43^. To investigate whether the diurnal cycle affects the *PnAlaAT*s, diurnal changes of expression level in leaves during a day/night cycle were determined. *PnAlaAT1* and *PnAlaAT2* expression fluctuated in different leaves during the diurnal cycle, and had the same periodicity (Fig. 7). *PnAlaAT3* and *PnAlaAT4* showed a similar expression pattern in leaves, with low fluctuation and expression levels during the diurnal cycle. In addition, the expression levels of *PnAlaAT1* were clearly higher than that of *PnAlaAT2*.

**Figure 8:**
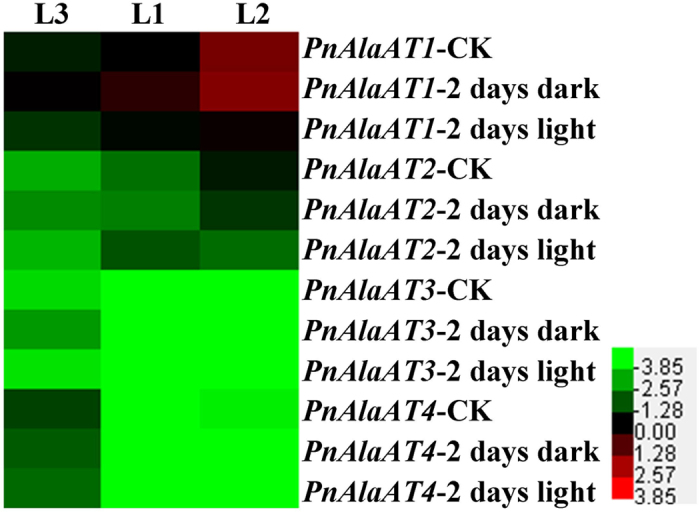
Light effects on the expression patterns of *PnAlaAT* genes in leaves. Plants were grown in darkness for 2 days, or grown in light for 2 days. Quantitative RT-PCR was performed using total RNA extracted from leaves.

To evaluate the effect of light induction on *PnAlaAT* gene family members, the transcript levels in plants kept for 2 days in the dark or 2 days in the light were examined (Fig. 8). The mRNA levels of *PnAlaAT1* increased significantly in L1 and L3 after 2 days of continuous dark, and the mRNA abundance of *PnAlaAT1* was highest in L2 after this treatment. The mRNA level of *PnAlaAT2* was lower than that of *PnAlaAT1* in all tested sections, and didn’t change significantly except in L2. *PnAlaAT3* and *PnAlaAT4* showed low expression levels in all conditions.

### Effects of MSX on *PnAlaAT*s expression in *P. simonii* × *P. nigra* roots

**Figure 9:**
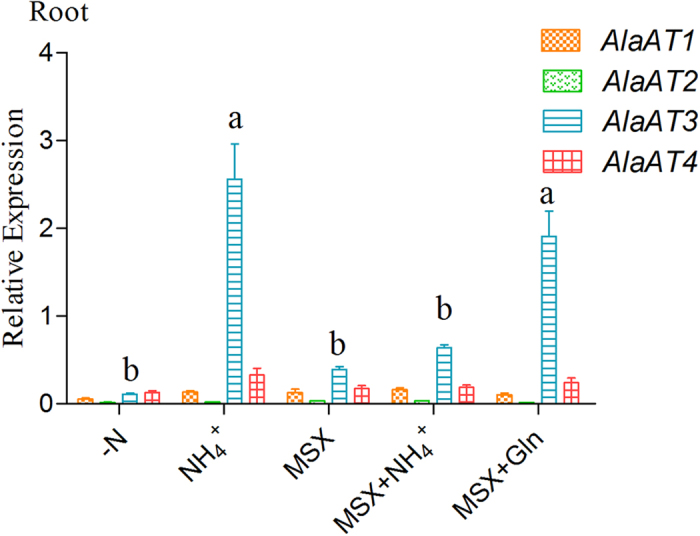
Effects of ammonium, glutamine and MSX on expression of *PnAlaAT* genes in roots. Plants were supplied with nitrogen-free medium for 4 days, after that plantlets were fertilized with different nutrient solutions, nitrogen-free nutrient solution (−N); nitrogen-free nutrient solution plus 10mM NH_4_Cl (+NH_4_^+^); nitrogen-free nutrient solution plus MSX (+MSX); nitrogen-free nutrient solution plus 10 mM NH_4_Cl and MSX (MSX+NH_4_^+^); nitrogen-free nutrient solution plus 10 mM Gln and MSX (MSX + Gln). Quantitative RT-PCR was performed using total RNA extracted from roots. Results are the mean ± SE of three replicates. (*P* < 0.05).

To clarify whether the expression of *PnAlaAT3* is dependent on NH_4_^+^ or glutamine or its related metabolites, MSX was applied to inhibit GS activity. GS functions in the glutamine synthetase/glutamine: α-oxoglutarate aminotransferase cycle (GS/GOGAT cycle) to generate glutamine from NH_4_^+^ and glutamate^44^. MSX blocks the enzyme activity of GS and prevents glutamine synthesis. NH_4_^+^ application followed the N starvation increased *PnAlaAT3* mRNA levels significantly in roots (Fig. 9). However, *PnAlaAT*3 expression showed no significant change when MSX was applied alone or with NH_4_^+^. In contrast, *PnAlaAT*3 mRNA levels were significantly induced by MSX with Gln. These treatments had no impact on *PnAlaAT1, PnAlaAT2* and *PnAlaAT4*. This suggested that glutamine rather than NH_4_^+^ itself controlled the expression of *PnAlaAT3* gene in roots.

## Discussion

The present work is the first report of *AlaAT* homologues in *Populus*. We identified four *AlaAT* genes in *P. trichocarpa*, namely *PtAlaAT1, PtAlaAT2, PtAlaAT3* and *PtAlaAT4*. The four genes were classified into two subgroups (Fig. 1) based on comparison with the sequences of *AlaAT* genes from *A. thaliana, G. max* and *M. truncatula*^22^. Subgroup A contained *PtAlaAT3* and *PtAlaAT4*, which were closely related to *A. thaliana* alanine aminotransferase (*AtAlaAT1* and *AtAlaAT2*), *G. max* alanine aminotransferase (*GmAlaAT1* and *GmAlaAT4*) and *M. truncatula* alanine aminotransferase (*MtmAlaAT*); subgroup B contained *PtAlaAT1* and *PtAlaAT2*, which were closely related to *A. thaliana* glutamate:glyoxylate aminotransferase (*AtGGAT1* and *AtGGAT2*), *G. max* alanine aminotransferase (*GmAlaAT2* and *GmAlaAT3*) and *M. truncatula* alanine aminotransferase (*MtcAlaAT*). According to Tuskan *et al*.^45^, some segments on chromosomes I and III and chromosomes VIII and X are presumed to have arisen from the salicoid-specific genome duplication. *PtAlaAT1–4* are located in these duplicated segments. This indicates that the two members of each subgroup might derive from a duplication event.

Many metabolic processes occur in leaves, such as synthesis of organic compounds, photosynthesis and photorespiration. Several studies have shown the N concentration of leaves generally decreases with increasing plant age^46^. In the chaparral shrub *Lepechinia calycina* growing in its natural habitat, photosynthetic capacity, leaf N content and stomatal conductance decreased with increasing leaf age^47^. In *Portulaca oleracea* L., the absolute amount of both ribulose bisphosphate carboxylase/oxygenase (rubisco) and phosphoenolpyruvate carboxylase was lower in senescent leaves than in mature leaves, and rubisco activity was reduced to a lesser degree^48^. In *Nicotiana tabacum*, metabolic, biochemical and molecular events occur during leaf ageing, with a particular emphasis on N metabolism. The sink/source transition also occurs at a particular leaf stage^49^. Additionally, the concentration of N supplied has an effect on leaf senescence^50^. On the basis of these results, we took leaf development and senescence into account. In our test conditions, we observed that the lower, old leaves wilted first, the uppermost, younger leaves expanded gradually and the middle leaves remained active for a long time. We believed these three sections of leaves represented different developmental periods, though the mechanism of leaf development is unclear. We therefore divided the leaves into three groups in our study.

*A. thaliana* has four *AlaAT* homologues. *AtAlaAT1* and *AtAlaAT2* encode alanine aminotransferase (E.C.2.6.1.2), whereas *AtGGAT1* and *AtGGAT2* contain peroxisome-targeting signal (PTS) sequences and have glutamate:glyoxylate aminotransferase activity (GGAT, E.C.2.6.1.4)^18^,^36^. PTSs were also found in PnAlaAT1 and PnAlaAT2 (Supplementary Figure 1). To reveal the biological roles of the *PnAlaAT* genes, their expression profiles were precisely analyzed. *PnAlaAT1* was expressed in all organs, with very high levels in leaves. Similarly, *PnAlaAT2* was expressed mainly in leaves, with the highest level in L3, but was negligibly expressed in stems and roots. In *A. thaliana*, the expression of *AtGGAT1* and *AtGGAT2* was much higher in green leaves than in other organs, but the *AtGGAT2* mRNA level was lower than that of *AtGGAT1* in all organs^18^. The very high similarity of *PnAlaAT1*/*PnAlaAT2* to *AtGGAT1*/*AtGGAT2* indicated that they might be peroxisomal proteins and should have the same function in the photorespiratory process, catalyzing the reaction of glutamate and glyoxylate to α-oxoglutarate and glycine. However, it needs to be further confirmed. Given the expression pattern of *PnAlaAT1* in response to light induction (Fig. 8), it seems interesting that *PnAlaAT1* expression was higher at night, while photorespiration only happens during the daytime. It is possible that *PnAlaAT1* is highly expressed at night and its products Gly would be used during the subsequent day. The further studies were needed to examine this hypothesis.

*PnAlaAT1* expression in L1 was affected by different NO_3_^−^ concentrations, but different NH_4_^+^ concentrations did not cause a significant change (Fig. 5). NO_3_^−^ reduction is related to photorespiration, which is the light-stimulated oxidation of photosynthesis intermediates to CO_2_^51^. This process occurs primarily in higher plants that fix CO_2_ via the C3 pathway of photosynthesis. Photorespiration protects C3 plants from photooxidation^52^ and occurs in the chloroplast, peroxisome and mitochondrion. In the peroxisome, glutamate:glyoxylate aminotransferase catalyzes the reaction of glutamate and glyoxylate to α-oxoglutarate and glycine^53^. Two isoforms exist in *A. thaliana*, with GGAT1 representing the major form in leaves^18^,^36^. Photorespiration stimulates provision of a reductant source for nitrate reductase^51^. Most NO_3_^−^ is reduced in leaves^54^ and is supplied to L1 predominantly. Additionally, NO_3_^−^ is considered not only a major macronutrient, but also a powerful signal molecule. NO_3_^−^ triggered signals can be rapidly and specifically sensed by plant cells and then the expression of a large set of genes regulating plant metabolism and growth are induced or inhibited^55^. In our results, *PnAlaAT1* expression in L1 was affected by different NO_3_^−^ concentrations and reached a peak after 12 h of 1 mM NO_3_^−^ supply. However, this kind of response didn’t occur in other organs. Based on the above results, we speculated that photorespiration in L1 was affected predominantly when NO_3_^−^ was supplied.

The expression level of *PnAlaAT1* and *PnAlaAT2* exhibited a diurnal fluctuation in leaves (Fig. 8). This periodic fluctuation may be controlled by an endogenous circadian clock, whose phase can be entrained by light, possibly through the phytochrome system^42^,^56^. The presence of putative light-regulation and circadian elements in the promoter regions of *PnAlaAT1* and *PnAlaAT2* is consistent with our data and may partially explain the expression patterns of these genes in leaves^57^,^58^,^59^. Previous studies showed that both AlaAT and GGAT activities were present in etiolated wheat seedlings but their activity was half of that observed in light-grown seedlings, and exposure of etiolated seedlings to light caused an increase in enzyme activities and upregulated *GGAT1* gene, while *AlaAT1* gene didn’t respond^60^. But in our study, the expression of *PnAlaAT1* and *PnAlaAT2* exhibited a diurnal fluctuation in leaves (Fig. 8) and *PnAlaAT1* increased significantly in L1 and L3 after 2 days of continuous dark (Fig. 9), while *PnAlaAT3* and *PnAlaAT4* didn’t exhibit these characteristics. The regulatory mechanism of *PnAlaAT1* and *PnAlaAT2* need to be further studied. Additionally, *OsAlaAT1* plays an essential role in the regulation of starch storage in rice endosperm^61^. This is consistent with our finding that endosperm expression elements existed in the promoter regions of the *PnAlaAT* genes.

The expression of *AlaAT* genes was diverse in different species. In soybean, *GmAlaAT1* and *GmAlaAT4* were expressed only in the roots of non-nodulated plants, with very low expression in the roots of nodulated plants^37^. In *M. truncatula, m-AlaAT* was expressed at very similar levels in roots, stems and leaves of adult plants and in the embryo axes of young seedlings^22^. In our study, *PnAlaAT3* and *PnAlaAT4* were expressed in all organs, while *PnAlaAT3* was expressed at a much higher level in roots than in the other organs (Fig. 4).

NO_3_^−^ and NH_4_^+^ are absorbed by roots through NRT and AMT, respectively^6^,^7^. NO_3_^−^ is reduced to NH_4_^+^ by NR and NiR, and then enters the glutamine synthetase (GS) and glutamate synthase (GOGAT) cycle. NH_4_^+^ is mainly assimilated in roots, whereas most NO_3_^−^ reduction occurs in the leaves of poplars^54^. Additionally, N concentration affects the NH_4_^+^ content and NR activities in poplar roots^62^. It has been confirmed that GS activity in roots is promoted by ammonium^63^. Ammonium has been identified as a signaling molecule^64^. In our study, *PnAlaAT3* expression in roots was increased more than 100 times after treatment with 10 mM NH_4_^+^ (Fig. 6). To determine whether this was due to the effect of the ammonium signal or the promotion of GS activity, we designed an experiment in which the GS activity was inhibited.

Glutamine, one of the N-assimilation products, can be synthesized from ammonium and glutamate by GS. Glutamine is the main transportable form of organic N and is a N-storing compound in plants^44^. As a major amino donor for the synthesis of amino acids and other N-containing compounds, glutamine can be taken up from the soil^65^. In addition to its role in nutrition and metabolism, glutamine can also function as a signal molecule inducing the expression of at least 35 genes involved in metabolism, transport, signal transduction, and stress responses within 30 min in rice^66^. Can Gln induce the expression of *AlaAT* genes? We showed in this study that Gln affected the expression level of *PnAlaAT3* in roots, but not the other three *PnAlaAT* genes. That is, NH_4_^+^ participated in the GS/GOGAT cycle to synthesize glutamine after being absorbed by the roots, and then glutamine or its related metabolites induced the expression of *PnAlaAT3. PnAlaAT1, PnAlaAT2* and *PnAlaAT4* genes were not significantly influenced by NH_4_^+^ or Gln in roots. In conclusion, *PnAlaAT3* was expressed at a higher level than other *PnAlaAT* genes in roots, and only the expression of *PnAlaAT3* was promoted by NH_4_^+^ or Gln or its related metabolites in roots. These results suggest that *PnAlaAT3* might play an important role in nitrogen metabolism.

In our previous study, we found that long-term application of different forms of nitrogen may cause morphological changes of poplar roots. However, we did not find significantly differentially expressed genes related to N metabolism pathway, mitochondrial electron transport/ATP synthesis and mineral nutrition in our previous report of global gene expression analysis utilizing RNA-seq. On the contrary, we found that the significantly differentially expressed genes are largely associated with fermentation, glycolysis, and tricarboxylic acid cycle (TCA), secondary metabolism, hormone metabolism and transport processing^67^. In the study of Beatty *et al*.^33^, the alanine aminotransferase (AlaAT) gene was transferred into rice plants and ectopically expressed under the control of a tissue-specific promoter to investigate their functions in uses of nitrogen sources. Consistent with our findings, the authors found the transgenic plants displayed a strong N use efficiency but less changes in the transgenic transcriptome compared with the controls, with only 0.11 and 0.07% differentially regulated genes in roots and shoots, respectively. We speculate that N metabolism related genes might play an important role in the regulation of short-time N metabolism, and affect morphology changes of poplar roots through regulating fermentation, glycolysis and tricarboxylicacidcycle (TCA), secondary metabolism, hormone metabolism and transport processing.

## Methods

### Tissue culture and growth of plants

Young leaves were collected from cuttings of *P. simonii* × *P. nigra* grown at Northeast Forestry University Forest Farm, Harbin, China. Explants were surface sterilized with 70% absolute ethyl alcohol for 1 min and 0.5% NaOCl (Purui, Shanghai, China) solution for 7 min, and then rinsed three times with sterile double-distilled water. The leaves were cut into squares (1 cm^2^). The leaf squares were cultivated in Petri dishes (diameter 9 cm) on MS medium^68^ with 0.5 mg/L 6-benzyl-aminopurine (PhytoTechnology, Lenexa, USA) and 0.05 mg/L β-naphthaleneacetic acid (PhytoTechnology, Lenexa, USA), shoots were induced on MS medium with 0.1 mg/L 6-benzyl-aminopurine and 0.05 mg/L β-naphthaleneacetic acid, and roots were induced on MS medium with 0.2 mg/L indole-3-butyric acid (PhytoTechnology, Lenexa, USA). When they reached a height of 10 cm, the plantlets were transferred to a greenhouse with a photosynthetic photon flux density (PPFD) of 100 μmol m^−2^ s^−1^, a 16-h-light/8-h-dark photoperiod, and a temperature of 22 °C. There were 114 plants from the hybrids of *P. simonii* × *P. nigra* were studied in this research and 546 samples (leaves, stems and roots) were collected for all the analysis.

### Nitrogen and inhibitor treatment

**Figure 10:**
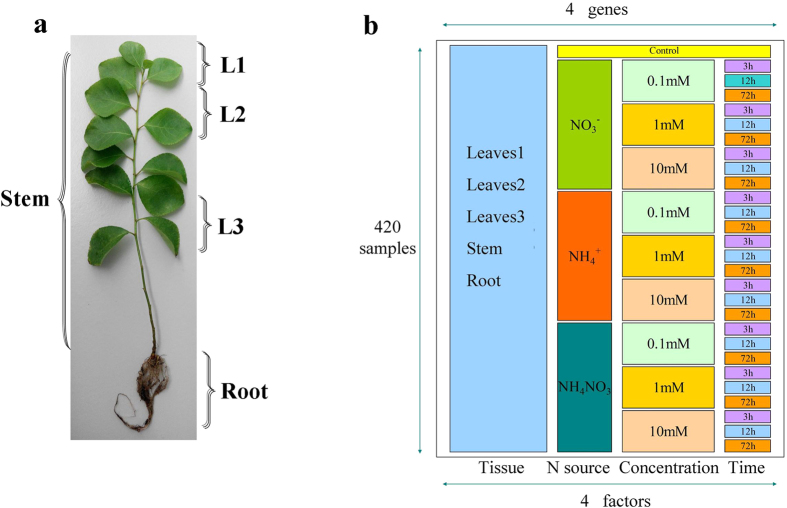
Scheme of the treatments and harvest for expression analysis of *PnAlaAT* genes. (**a**), distribution of different organs in *P. simonii × P. nigra*, leaves: 1^st^–3^rd^ (L1) and 4^th^–6^th^ (L2) are the ones from the top of the plants, 1^st^-3^rd^ (L3) are the ones from the bottom of the plants. (b), the four factors (tissue, N source, concentration and time). The control was treated with nitrogen-free nutrient solution for 0 h. There were 420 samples in total.

Each plant was grown in one plastic pot filled with sterilized vermiculite as a substrate and supplied with 200 mL of sterile modified Long-Ashton nutrient solution (1 mM nitrogen)^68^ every 2 days. The treatments were performed when the seedlings were 30 cm tall. The plants were supplied with 200 mL of sterile N-free nutrient solution for 4 days. The effects of different N sources on the plants were then tested by adding NH_4_Cl or NaNO_3_ or both to the N-free nutrient solution to final concentrations of 0.1, 1 and 10 mM. To avoid changing the osmotic pressure, 0.5 mM KCl and 0.9 mM CaCl_2_ (Bodi, Tianjin, China) were used (Table S1)^69^. The plant tissues (1^st^–3^rd^ (L1) and 4^th^–6^th^ (L2) leaves from the top of the plant, 1^st^–3^rd^ leaves (L3) from the bottom of the plant, stems and roots) were harvested after 0 h (control), 3 h, 12 h and 72 h treatment (Fig. 10). The plants were treated at different times, however, at the same time harvested at 11 o’clock. Three repeated samples were frozen in liquid nitrogen and stored at −80 °C for further analysis.

To examine the effect of methionine sulfoximine (MSX, an inhibitor of glutamine synthase), we supplied various test nutrient solutions (N-free nutrient solution; N-free nutrient solution plus 10 mM NH_4_Cl; N-free nutrient solution plus 1 mM MSX; N-free nutrient solution plus 10 mM NH_4_Cl and 1 mM MSX; N-free nutrient solution plus 10 mM Gln and 1 mM MSX). The plant seedlings were cultivated in nitrogen-free nutrient solution for 4 days, and then were transferred to the five aforementioned culturing solutions, respectively. After 12 h treatment, plant samples were harvested, frozen in liquid nitrogen immediately, and stored at −80 °C for future analysis.

### Light treatment

Plantlets grown in tissue culture vessels were directly transferred to soil supplied with water every 2 days. To examine the influence of the diurnal cycle, samples were harvested every 3 h over one day. To examine the effect of light, the plants were grown in darkness for 2 days and then transferred to a 16-h-light/8-h-dark photoperiod for 4 days. Three repeated samples were frozen in liquid nitrogen and stored at −80 °C for analysis.

### Identification of AlaAT gene family members in P. trichocarpa

We downloaded the Hidden Markov Model (HMM) profile file (Aminotran_1_2.hmm) of the Pfam Aminotran_1_2 domain (PF00155) from the Pfam database^70^. The protein sequences of *P. trichocarpa* were downloaded from Phytozome 9.0 (http://www.phytozome.net/poplar_er.php). We used the HMM modules of PF00155 with the HMMER (v 3.0) software to search the proteome of *P. trichocarpa*^71^. Proteins with e-values less than 2.2E-34 were included in further analyses. We searched the Aminotran_1_2-domain in all the collected proteins using the Interproscan (http://www.ebi.ac.uk/Tools/pfa/iprscan/) and SMART software^72^. We used the Gene Structure Display Server (GSDS) program to illustrate the exon/intron organization of individual *AlaAT* genes^73^.

### RNA extraction and reverse transcription

Total RNA was extracted from leaf, stem, and root tissues using the pBIOZOL plant total RNA Extraction Reagent (BioFlux, Hangzhou, China) according to the manufacturer’s protocol. The integrity of the RNA was verified by 1.5% agarose gel electrophoresis. After removing genomic DNA with gDNA Eraser, approximately 2 μg RNA was used to synthesize the first-strand cDNA using the PrimerScript RT Reagent Kit (Takara Biotechnology, Dalian, China).

### Real-time PCR (RT-PCR)

The Primer Premier 5.0 (Premier Biosoft, Palo Alto, CA, USA) software was used to design specific primers for real-time PCR analysis and the primer sequences of each gene were included in Table 2. The following gene-specific primers were used: for *AlaAT1*. Real-time PCR was performed using a 7500 Real-Time PCR System (Applied Biosystems) with SYBR Green PCR Master Mix (Applied Biosystems) according to the manufacturer’s protocol. Each reaction was performed on 5 μL of a 1:5 (v/v) dilution of the first-strand cDNA, synthesized as described above, with 0.3 μM of each primer in a total reaction of 20 μL. The specificity of the PCR amplification procedures was checked with a heat dissociation protocol after the final cycle. The amplification program had three steps: (1) 1 cycle (95 °C, 10 min); (2) 40 cycles, cDNA denaturing (95 °C, 15 s), hybridization and extension (60 °C, 1 min); (3) 1 cycle (95 °C, 15 s; 60 °C, 1 min; 95 °C, 15 s; 60 °C, 15 s) to generate a dissociation curve to confirm the specific amplification of each individual reaction. Each reaction was done in triplicate and the corresponding Ct values were determined. In the expression analyses, transcript levels were normalized to the *PtActin2* gene (accession number: XM_002298946) as it is expressed stability independently of tissues, N sources, N concentration and developmental stage. The 2^−ΔΔCT^ method was used to analyze the relative changes in gene expression^74^,^75^.

### Promoter analysis

Regulatory elements in the 5′-upstream regions of the poplar *AlaAT* genes were predicted starting from the ATG codon for initiation of translation. Sequence identity was analyzed to identify putative cis-acting elements using the PlantCARE database^76^. Sequence stretches of 1500 bp for each gene were compared.

### Statistical analysis

Statistical tests were performed with SPSS 19.0 (IBM, USA), and the data were tested to confirm their normality before statistical analysis. For experimental variables, one-way analysis of variance (ANOVA) was used with N-treatment as a factor. Differences between means were considered significant when *P *< 0.05 according to the ANOVA *F*-test.

Our work demonstrated that the poplar genome contained four genes encoding alanine aminotransferase (*PnAlaAT3* and *PnAlaAT4*) and glutamate:glyoxylate aminotransferase (*PnAlaAT1* and *PnAlaAT2*). *PnAlaAT1* and *PnAlaAT2* were closely related to *AtGGAT1* and *AtGGAT2*, and contained PTS1-like sequences in their proteins. They were expressed predominantly in leaves and induced by NH_4_^+^ and NO_3_^−^. Their expression exhibited a diurnal fluctuation in leaves. The expression level of *PnAlaAT1* was higher than that of *PnAlaAT2* in all conditions. *PnAlaAT3* and *PnAlaAT4* were expressed in roots, stems and leaves. The expression level of *PnAlaAT3* was higher than that of *PnAlaAT4. PnAlaAT3* expression was increased significantly by NH_4_^+^ in roots, because of Gln or its related metabolites. We speculated that *PnAlaAT1* and *PnAlaAT3* might play important roles in leaves and roots, respectively. These results offered new insight into the *AlaAT* gene family in woody plants and the involvement of *AlaAT* genes in woody plant responses to exogenous N and light.

## Additional Information

**How to cite this article**: Xu, Z. *et al*. Identification and expression analyses of the alanine aminotransferase (AlaAT) gene family in poplar seedlings. *Sci. Rep.* **7**, 45933; doi: 10.1038/srep45933 (2017).

**Publisher's note:** Springer Nature remains neutral with regard to jurisdictional claims in published maps and institutional affiliations.

## Supplementary Material

Supplementary Data

## Figures and Tables

**Table 1:** AlaAT gene family in *Populus trichocarpa*.

S.N	Name	Accession Number	Chromosome Location	ORF(bp)	Protein Size	Exon Number	Location	E-value
1	*PtAlaAT1*	XM_002315639	Chr10:7600723–7606675	1446	481	13	62–461	4.8E-38
2	*PtAlaAT2*	XM_002312643	Chr08:12932980–12938537	1446	481	13	64–461	2.2E-140
3	*PtAlaAT3*	XM_006369021	Chr01:13606954–13612423	1446	481	16	80–468	1.6E-38
4	*PtAlaAT4*	XM_002304219	Chr03:10141011–10146425	1446	481	15	86–468	2.2E-34

**Table 2:** Details of primers used for polymerase chain reaction analysis.

Primer name	Sequences(from 5′to 3′)
*AlaAT1*- sense	GATCCAAATGTGGGGTTGCTATA
*AlaAT1*- antisense	CTGCTACCTTCCTAACTCCA
*AlaAT2*- sense	TATGAAGGCACGGTGGTTATT
*AlaAT2*- antisense	TTAGACGTTGGATTGAGCAGGT
*AlaAT3*- sense	CTTTTACTGTCGCAGCCTACTC
*AlaAT3*- antisense	GTTCAAGGCAACATCTTATTTTG
*AlaAT4*- sense	AGTTGTCTCCCGTCTCACAGAG
*AlaAT4*- antisense	CTTCGATGGAGGAGCAATAAAG
PtActin2- sense	CACAACTGCTGAACGGGAAAT
PtActin2- antisense	CAGGGCAACGGAAACACTCT
