# Supplementary material for: Identification and expression analyses of the alanine aminotransferase (AlaAT) gene family in poplar seedlings
Source: Sci Rep. 2017 Apr 5;7:45933. doi: 10.1038/srep45933 (PMC5380993; doi:10.1038/srep45933)
Supplement: Supplementary Data [file srep45933-s1.doc]

**Identification and expression analyses of the alanine aminotransferase (AlaAT) gene family in poplar seedlings**

**Zhiru Xu^1,2^, Jing Ma^2^, Chunpu Qu^1,3^, Yanbo Hu^2^, Bingqing Hao^1^, Yan Sun^1^, Zhongye Liu^1^, Han Yang^1^, Chengjun Yang^3^, Hongwei Wang^2^, Ying Li^4^, and Guanjun Liu^1,3,*^**

^1^ State Key Laboratory of Tree Genetics and Breeding (Northeast Forestry University), School of Forestry, Northeast Forestry University, Harbin 150040, China

^2^ College of Life Science, Northeast Forestry University, Harbin 150040, China

^3^ School of Forestry, Northeast Forestry University, Harbin 150040, China

^4^ Key Laboratory of Saline-alkali Vegetation Ecology Restoration in Oil Field (SAVER), Ministry of education, Alkali Soil Natural Environmental Science Center (ASNESC), Northeast Forestry University, Harbin 150040, China

**^*^**Corresponding. liuguanjun2013@nefu.edu.cn


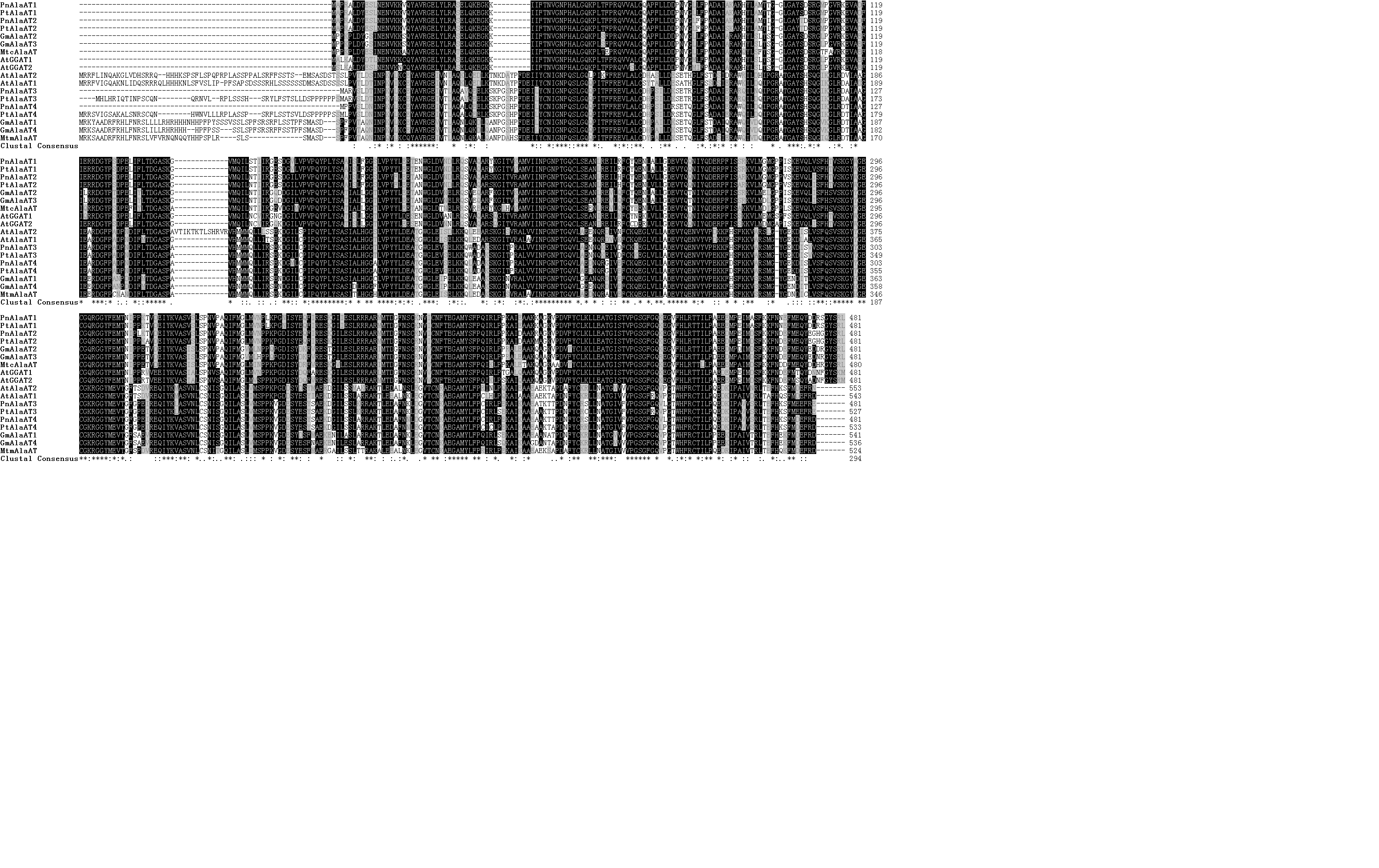


**Fig. S1.** Amino acid sequences alignment of *AlaATs* from *A. thaliana*, *M. truncatula*, *G. max*, *P. trichocarpa* and *P. simonii × P. nigra*.

**
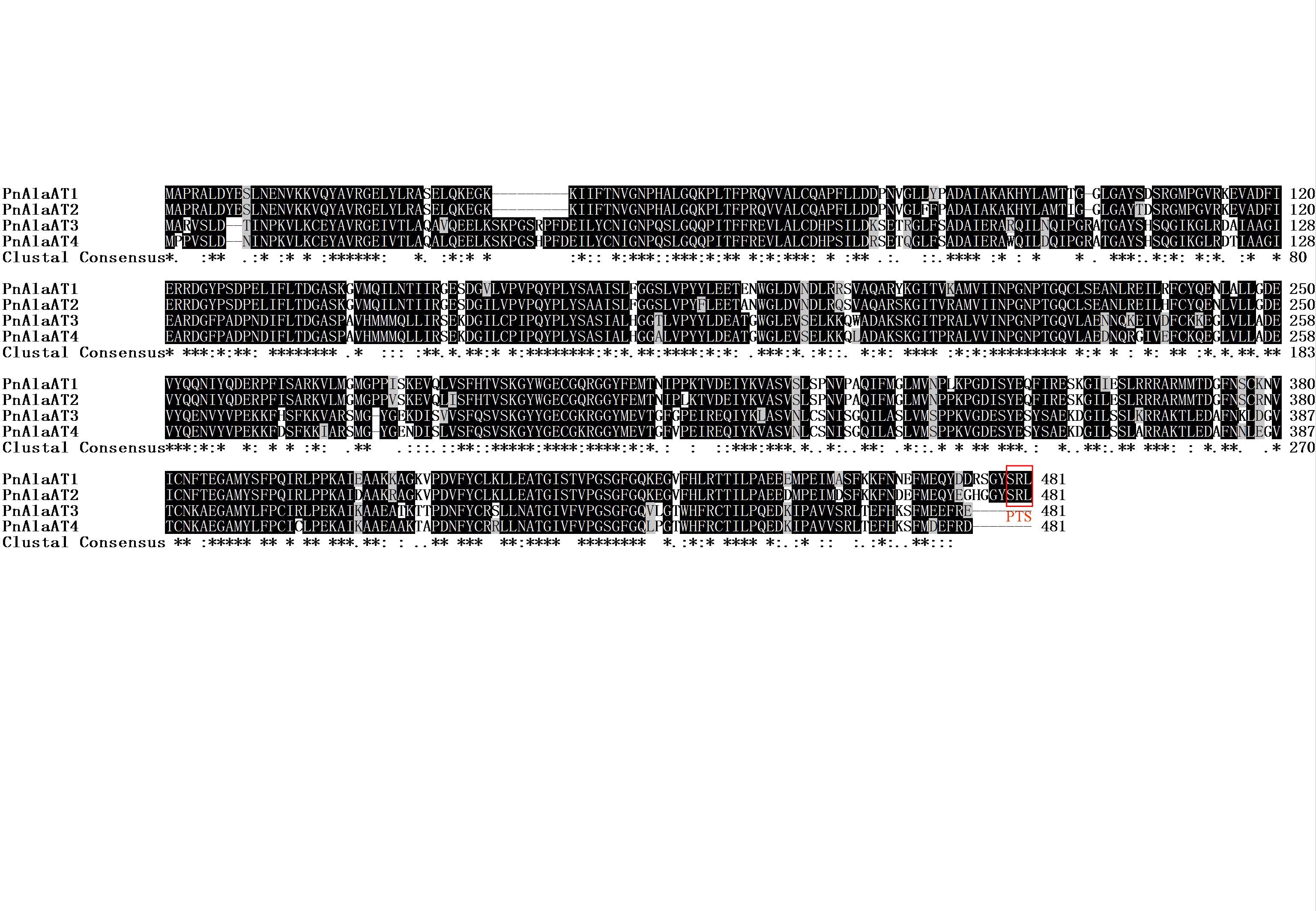
**

**Fig. S2.** Amino acid sequences alignment of the four AlaATs in *P. simonii×P. nigra*. Corresponding public locus numbers of the Alanine aminotransferases (PnAlaAT1-4) are XM_002315639, XM_002312643, XM_006369021 and XM_002304219.


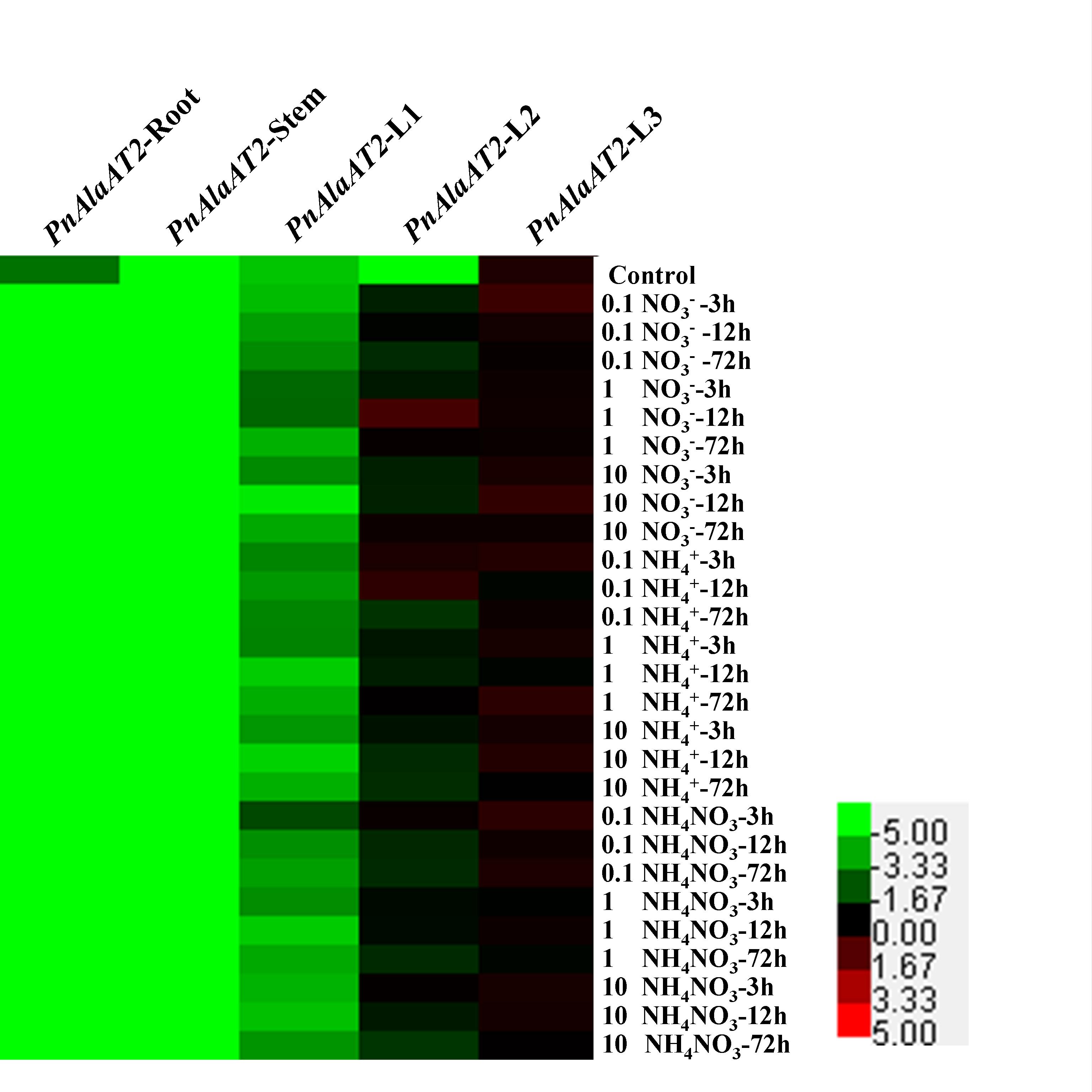


**Fig. S3.** Expression patterns of *PnAlaAT2* gene in different organs of *P. simonii × P. nigra* under different nitrogen source conditions. Leaves: 1^st^-3^rd^ (L1) and 4^th^-6^th^ (L2) are the ones from the top of the plants, and 1^st^-3^rd^ (L3) are the ones from the bottom of the plants. Quantitative RT-PCR was performed using total RNA extracted from leaves, stems and roots.


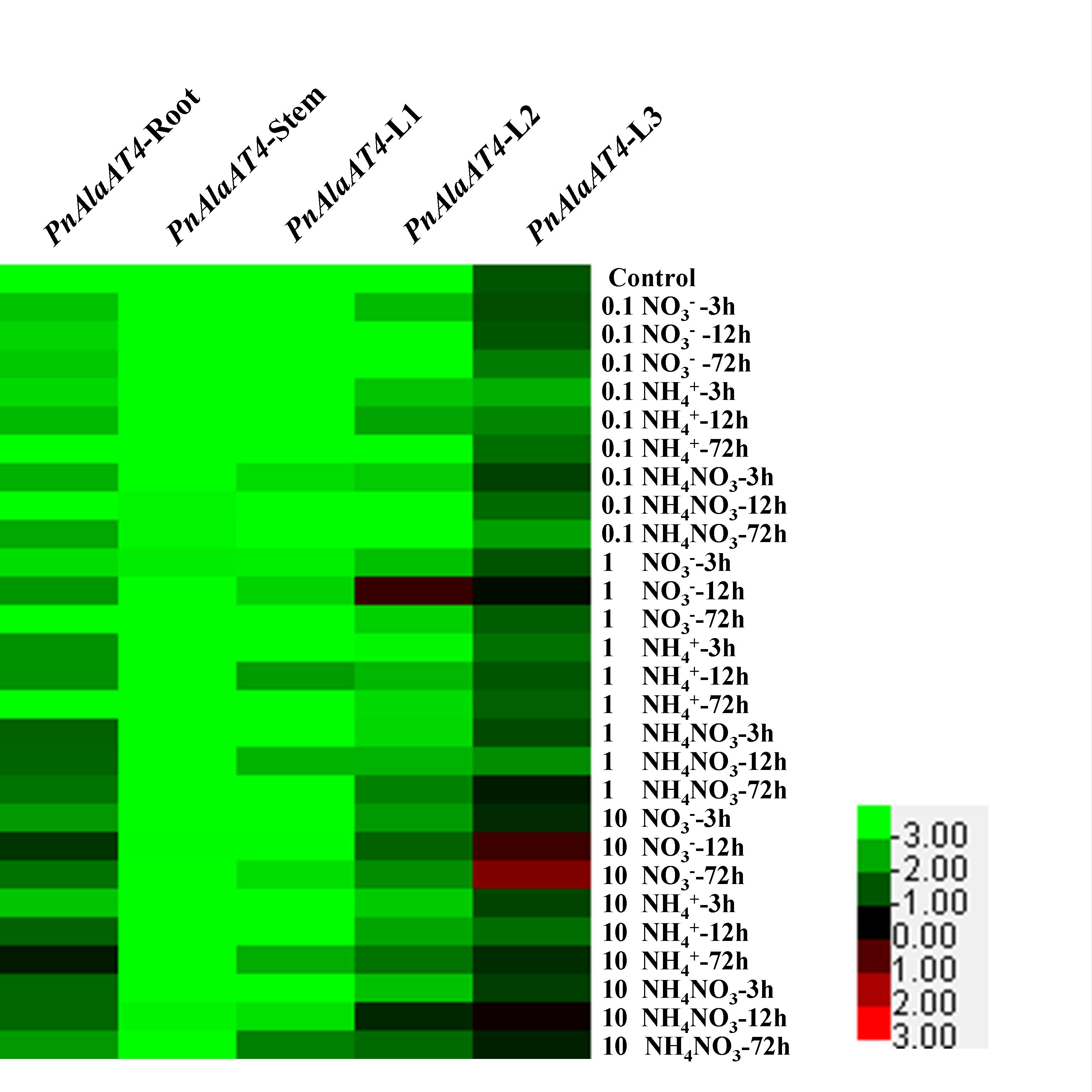


**Fig. S4.** Expression patterns of *PnAlaAT4* gene in different organs of *P. simonii × P. nigra* under different nitrogen source conditions. Quantitative RT-PCR was performed using total RNA extracted from leaves, stems and roots.

| Chemicals | mM |
| --- | --- |
| MnSO_4 `_H_2_O | 0.002 |
| H_3_BO_3_ | 0.01 |
| Na_2_MoO_4_ | 0.007 |
| CoSO_4_ | 0.00005 |
| ZnSO_4`_7H_2_O | 0.0002 |
| CuSO_4`_5H_2_O | 0.0002 |
| Na_2_EDTA | 0.01 |
| FeSO_4`_7H_2_O | 0.01 |
| MgSO_4_ 7H_2_O | 0.3 |
| KH_2_PO_4_ | 0.6 |
| K_2_HPO_4`_3H_2_O | 0.042 |
| KCl | 0.5 |
| CaCl_2_ | 0.9 |

**Table S1** Modified Long-Ashton medium.
